# Ni-Modified Ag/SiO_2_ Catalysts for Selective Hydrogenation of Dimethyl Oxalate to Methyl Glycolate

**DOI:** 10.3390/nano12030407

**Published:** 2022-01-26

**Authors:** Shuai Cheng, Tao Meng, Dongsen Mao, Xiaoming Guo, Jun Yu, Zhen Ma

**Affiliations:** 1 School of Chemical and Environmental Engineering, Shanghai Institute of Technology, Shanghai 201418, China; 156061301@mail.sit.edu.cn (S.C.); guoxiaoming@sit.edu.cn (X.G.); yujun@sit.edu.cn (J.Y.); 2 Department of Environmental Science and Engineering, Fudan University, Shanghai 200438, China; zhenma@fudan.edu.cn

**Keywords:** dimethyl oxalate, methyl glycolate, selective hydrogenation, nickel modification, Ag/SiO_2_

## Abstract

Ni-modified Ag/SiO_2_ catalysts containing 0~3 wt.% Ni were obtained by impregnating Ni species onto Ag/SiO_2_ followed by calcination and reduction. The catalysts’ performance in the hydrogenation of dimethyl oxalate (DMO) to methyl glycolate (MG) was tested. Ag-0.5%Ni/SiO_2_ showed the highest catalytic activity among these catalysts and exhibited excellent catalytic stability. The effects of the Ni content on the structure and surface chemical states of catalysts were investigated by XRF, N_2_-sorption, XRD, TEM, EDX-mapping, FT-IR, H_2_-TPR, UV–vis, and XPS. The better catalytic activity and stability of Ni-modified Ag/SiO_2_ (versus Ag/SiO_2_) are ascribed to the improved dispersion of active Ag species as well as the higher resistance to the growth of Ag particles due to the presence of Ni species.

## 1. Introduction

Methyl glycolate (MG), containing both hydroxyl and ester groups, is an important fine chemical [1,2,3]. As it has similar chemical properties to alcohol and ester, MG can undergo various reactions such as carbonylation, hydrolyzation, and oxidation [4,5]. Many traditional synthetic technologies, such as the carboxylation of formaldehyde and condensation of methyl formate with formaldehyde [2,6,7], have been applied for the synthesis of MG. However, disadvantages such as environmental pollution, harsh production process, and low stability of catalyst are obvious [2,6,7]. Thus, the development of a green synthetic method with high efficiency is warranted. The synthesis of MG by catalytic hydrogenation of dimethyl oxalate (DMO) has been proposed as a more economical and environmentally friendly route compared with other catalytic procedures [2,3,8,9,10].

The hydrogenation of DMO contains the successive hydrogenation of DMO to MG, MG to ethylene glycol (EG), and EG to ethanol (EtOH) [11]. To make sure that the hydrogenation of DMO stops at the formation of MG, catalysts with relatively weak hydrogenolysis ability should be used. Ag-based catalysts such as Ag/MCM-41 [12], Ag/SBA-15 [13], Ag/CNT [14], Ag/KCC-1 [15,16], Ag/SiO_2_ [17], Ag/AC-N [18], and Ag/AS [7] have been reported for that purpose. However, the catalytic activity and stability should be improved [19,20] in order to satisfy industrial demands and practical application.

The catalytic properties and stability of metallic catalysts can be improved by adding a promoter [21,22,23,24]. The promoted catalysts can exhibit superior catalytic performance in many reactions such as catalytic reforming [21], selective oxidation of alcohols [22], and selective hydrogenation [23,24]. Recently, several Ag-containing promoted catalysts were applied in the MG synthesis from selective hydrogenation of DMO [5,19,25]. For instance, Zheng et al. [25] demonstrated that Au-Ag/SBA-15 showed high efficiency and revealed that the changes of particle dispersion and electronic structure of metal particles promote the activity. Li et al. [19] and Zhou et al. [5] reported that both the catalytic activity and stability of Ag-Ni/SBA-15 were higher than those of Ag/SBA-15. It is known that silica sol is cheaper than SBA-15, and the nanoscale SiO_2_ particle is highly dispersed, so it is a good support for loading metal components. Recently, our group found that the 1 wt.% Ni modified 10% Ag/SiO_2_ prepared by a single-step ammonia-evaporation deposition-precipitation method exhibited lower activity and selectivity than 10% Ag/SiO_2_ [26].

Herein, we report Ag-Ni/SiO_2_ catalysts with several Ni loadings for the vapor-phase hydrogenation of DMO to MG. The catalysts were synthesized by ammonia evaporation with cheap silica sol followed by simple impregnation of an aqueous Ni(NO_3_)_2_ solution. The preparation method is different from that in our previous work [26] and leads to better catalytic performance. The effects of Ni loadings on the physicochemical properties of Ag-Ni/SiO_2_ were investigated via comprehensive characterization, and the structure–activity correlation was also discussed.

## 2. Experimental

### 2.1. Materials

The analytical grade chemical reagents including AgNO_3_, Ni(NO_3_)_2•_6H_2_O, and aqueous ammonia solution were obtained from Adamas (Shanghai, China). The 30 wt.% silica sol was purchased from Qingdao Haiyang Chemicals (Qingdao, China). All the above reagents were used as received.

### 2.2. Preparation

Ag/SiO_2_ with a preset Ag loading of 10 wt.% was prepared by an ammonia-evaporation method [26]. First, a certain amount of AgNO_3_ was dissolved in 150 mL of deionized water. An aqueous ammonia solution was then added, and the mixture was stirred vigorously for 30 min at 60 °C. A certain amount of 30 wt.% silica sol was added, and the mixture was stirred vigorously for 4 h. The suspension, with an initial pH value of 11–12, was heated at 90 °C to allow for the evaporation of ammonia, the decrease in pH value, and the deposition of Ag species onto silica. The evaporation process was ended when the pH value was 7–8. The precipitate was washed with deionized water three times, and the filter cake was dried at 120 °C overnight to obtain Ag/SiO_2_ precursor (gray powders).

Ni-modified Ag/SiO_2_ precursors were prepared by impregnation. A calculated amount of Ni(NO_3_)_2•_6H_2_O was dissolved in 10 mL deionized water, then the Ni(NO_3_)_2_ solution was dropped onto 5 g Ag/SiO_2_ placed in a 100 mL crucible. The slurry was ultrasonically treated at room temperature for 30 min, dried at 120 °C overnight, and calcined in an oven at 350 °C under static air for 4 h, yielding Ni-modified Ag/SiO_2_. The heating rate before reaching 350 °C was 1 °C∙min^−1^. The samples were denoted as Ag-x%Ni/SiO_2_ in which x% represents the weight percentage of Ni. For the preparation of Ag-0.2%Ni/SiO_2_, Ag-0.5%Ni/SiO_2_, Ag-1%Ni/SiO_2_, and Ag-3%Ni/SiO_2_, the amounts of dissolved Ni(NO_3_)_2•_6H_2_O were 0.051, 0.128, 0.256, and 0.769 g, respectively. The method used for the preparation of 0.5%Ni/SiO_2_ was the same as that of Ag-0.5%Ni/SiO_2_ except that no AgNO_3_ was added in the ammonia-evaporation step.

### 2.3. Characterization

The chemical compositions of as-prepared samples were determined by using X-ray fluorescence spectroscopy (XRF, ZSX Priums, Rigaku, Tokyo, Japan).

The textural properties of the catalysts degassed under vacuum at 200 °C for 6 h were determined using N_2_ sorption at −196 °C on a Micromeritics ASAP 2020 HD88 apparatus (Norcross, GA, USA). The total pore volumes were derived from the adsorbed N_2_ volume at P/P_0_ = 0.99, and the pore size distributions and average pore diameters were derived based on the BJH method according to the desorption isotherms.

TEM images were recorded using a JEOL JEM 2100F transmission electron microscope (Tokyo, Japan) operated at an acceleration voltage of 200 kV. EDX-mapping experiments were conducted with a scanning TEM (STEM) mode.

IR spectra of translucent disks prepared by pressing powder samples dispersed in KBr (2 wt.%) were obtained on a Nicolet iZ10 FT-IR instrument (Thermo Fisher Scientific, Baltimore, MD, USA). The spectral resolution was 4 cm^−1^, and 32 scans were recorded in order to generate each spectrum.

Temperature-programmed reduction with H_2_ (H_2_-TPR) was conducted on a linear quartz micro-reactor in which 100 mg catalyst was pretreated at 200 °C for 2 h under N_2_. After being cooled down to room temperature, the catalyst was exposed to 10% H_2_/N_2_ mixture (30 cm^3^∙min^−1^), and the temperature of the catalyst bed was ramped to 650 °C at a rate of 10 °C∙min^−1^. The H_2_ consumption was recorded using a thermal conductivity detector (TCD).

XRD patterns were obtained on a PANalytical X’Pert instrument (Malvern, UK) using Ni-filtered Cu Kα radiation. The reduced catalysts were prepared in 10% H_2_/N_2_ (50 cm^3^∙min^−1^) at 300 °C for 2 h. The full width at half maximum of the Ag(111) peak at 38.1° was used to calculate the metal particle sizes based on the Scherrer equation.

UV–vis diffuse reflectance spectra (DRS) of the catalysts freshly reduced in 10% H_2_/N_2_ at 300 °C for 2 h were obtained on a UV–vis-NIR UV-3600 instrument (Shimadzu, Kyoto, Japan).

XPS data were obtained on an ESCALA 250 Xi spectrometer (Thermo Fisher Scientific, Baltimore, MD, USA) with a standard Al Kα X-ray source (1486.6 eV). The C 1s peak at 284.6 eV was used for calibration. The samples were reduced with 10% H_2_/N_2_ (50 cm^3^∙min^−1^) at 300 °C for 2 h before measurement. Afterwards, the samples were sent to the laboratory for the XPS measurements as soon as possible. The experimental error was given within ±0.1 eV.

### 2.4. Reaction Testing

The catalytic activity test was conducted on a continuous-flow, fixed-bed reactor. Typically, 0.5 g catalyst (40–60 mesh) was packed in between two layers of quartz sand in a stainless-steel tubular reactor. Prior to testing, the catalyst was activated at 300 °C (heating rate = 1 °C∙min^−1^) for 4 h in flowing pure H_2_ (50 cm^3^∙min^−1^) under atmospheric pressure. After the catalyst bed was cooled to the reaction temperature and the system pressure was maintained at 1.5 MPa, a methanol solution containing 13 wt.% DMO was injected by using a Series 2PB constant-flux pump. The products were analyzed with a Qiyang GC-9860 gas chromatograph fitted with a HP-INNOWAX capillary column and a flame ionization detector. The DMO conversion (*X_DMO_*) and product selectivity (*S_i_*) were calculated:

(1) $$X_{DMO}=(1-A_{DMO}f_{DMO}/\sum A_{i}f_{i})\times 100\%$$

(2) $$S_{i}=(A_{i}f_{i}/\sum A_{i}f_{i})\times 100\%$$

where *A_i_* and *f_i_* are the peak area and the molar correction factor of the individual component *i* in the product stream, respectively.

The turnover frequency (*TOF*, the moles of DMO converted per hour by each mole of surface Ag) was calculated [5]:

(3) $$TOF=\frac{C_{DMO}\times X_{DMO}\times V}{D\times N_{Ag}}$$

where *C_DMO_* is the DMO concentration in the DMO/methanol solution, *X_DMO_* is the DMO conversion, *V* is the flow rate of the DMO/methanol solution, *D* is the Ag dispersion based on the XRD analyses and *N_Ag_* is the total amount of Ag. The DMO conversion was limited to less than 30% to provide proper data for the TOF calculation by adjusting LHSV [13].

## 3. Results and Discussion

### 3.1. Characterization

The compositions of calcined catalysts were examined using XRF (Table 1). The actual Ag loading is close to the designed value (10 wt.%), signifying the effectiveness of the ammonia-evaporation method [26]. The actual amounts of Ni are slightly lower than the theoretical values, probably due to a partial loss of the Ni species during the catalyst preparation.

The textural properties of calcined catalysts were characterized. In Table 1, the BET surface areas of Ag-Ni/SiO_2_ catalysts range from 171.4 to 153.9 m^2^∙g^−1^, lower than that of Ag/SiO_2_ (179.4 m^2^∙g^−1^). The pore volume of Ag/SiO_2_ is 0.65 cm^3^∙g^−1^. The incorporation of Ni species slightly decreases the pore volume to 0.64–0.60 cm^3^∙g^−1^, whereas the pore diameters of Ag-Ni/SiO_2_ catalysts enlarged slightly. The decrease in the BET surface areas and pore volume of Ag-Ni/SiO_2_ catalysts may be due to the pore-clogging effect of the deposited Ni species.

**Figure 1:**
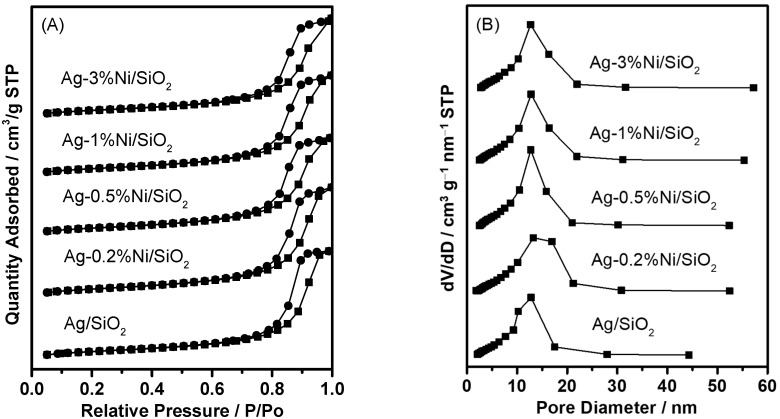
N_2_ sorption isotherms (**A**) and BJH pore size distributions (**B**) of the calcined catalysts.

The N_2_ sorption isotherms of calcined catalysts (Figure 1A) all exhibited Langmuir type IV isotherms with H1-type hysteresis loop, characteristic of nanostructured materials with uniform mesopores [27,28]. The BJH pore size distribution curves based on the desorption isotherms all displayed only one pore distribution peak (Figure 1B), indicating that the pore size distribution of each sample was concentrated, and the pore size was in the mesoporous range. Compared with Ag/SiO_2_, the larger pore could be observed in the Ni-containing catalysts, illustrating that the incorporation of Ni species probably covered some small pores [12].

**Figure 2:**
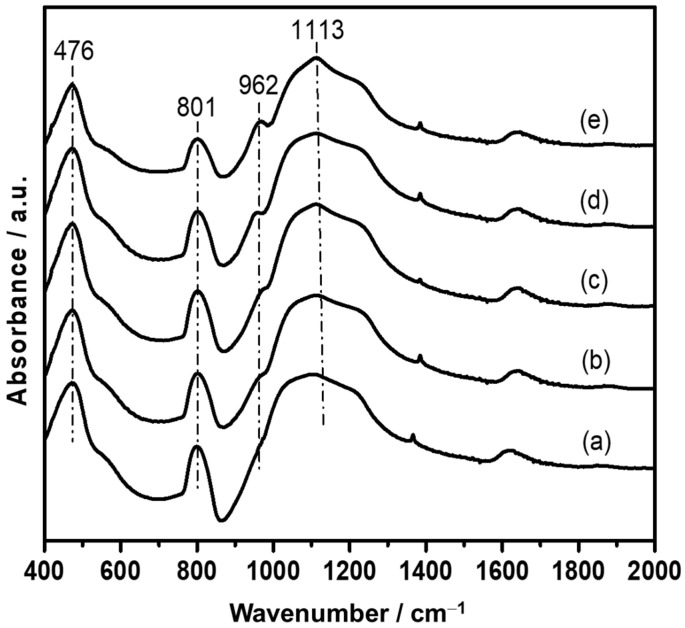
FT-IR spectra of calcined catalysts: (a) Ag/SiO_2_, (b) Ag-0.2%Ni/SiO_2_, (c) Ag-0.5%Ni/SiO_2_, (d) Ag-1%Ni/SiO_2_, and (e) Ag-3%Ni/SiO_2_.

The FT-IR spectra of the calcined samples were obtained to give more information. In Figure 2, all the samples have the adsorption bands at 1113, 801, and 476 cm^−1^, ascribed to the different vibration modes of the Si–O bonds of SiO_2_ [29]. Compared with Ag/SiO_2_, a new peak appeared at 962 cm^−1^ in Ag-Ni/SiO_2_. The band at 962 cm^−1^ can be assigned to Si–O vibrations of the Ni–O–Si group [30,31], since absorption bands characteristic of the metal–O–Si groups appear in the 900–1100 cm^−1^ region. Moreover, the relative intensity of the band at 962 cm^−1^ increased with the Ni content in the catalysts.

**Figure 3:**
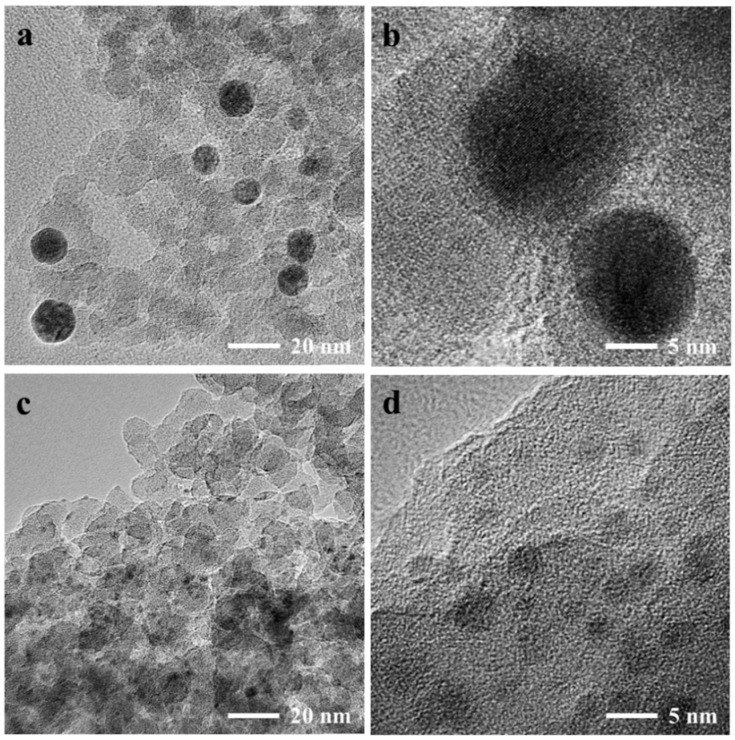
TEM images of the calcined Ag/SiO_2_ (**a**,**b**) and Ag-0.5%Ni/SiO_2_ (**c**,**d**).

**Figure 4:**
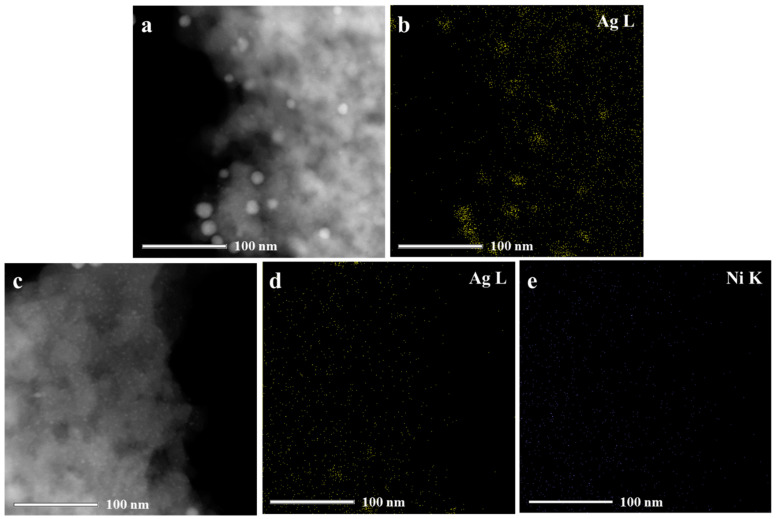
STEM and EDX-mapping images of calcined Ag/SiO_2_ (**a**,**b**) and Ag-0.5%Ni/SiO_2_ (**c**,**d**,**e**).

The morphologies of the representative catalysts were investigated by TEM (Figure 3). For both calcined Ag/SiO_2_ and Ag-0.5%Ni/SiO_2_, their SiO_2_ supports are irregular and the supported Ag species are spherical. Based on our group’s previous study [26], Ag species on calcined catalysts were mainly metallic Ag. The average Ag particle sizes on Ag/SiO_2_ (Figure 3a,b) are 10–20 nm. However, obviously smaller Ag particles of approximately 2–4 nm are obtained for Ag-0.5%Ni/SiO_2_ (Figure 3c,d). Furthermore, EDX-mapping was used to investigate the dispersion of Ag species (Figure 4 and Figure S1). Compared with Ag/SiO_2_ (Figure 4b), Ag species are better dispersed on Ag-0.5%Ni/SiO_2_ (Figure 4d). The above results indicated that the introduction of Ni could suppress the growth of Ag particles and promote the dispersion of Ag species on SiO_2_. In addition, the Ni species on Ag-0.5%Ni/SiO_2_ is also highly dispersed (Figure 4e).

**Figure 5:**
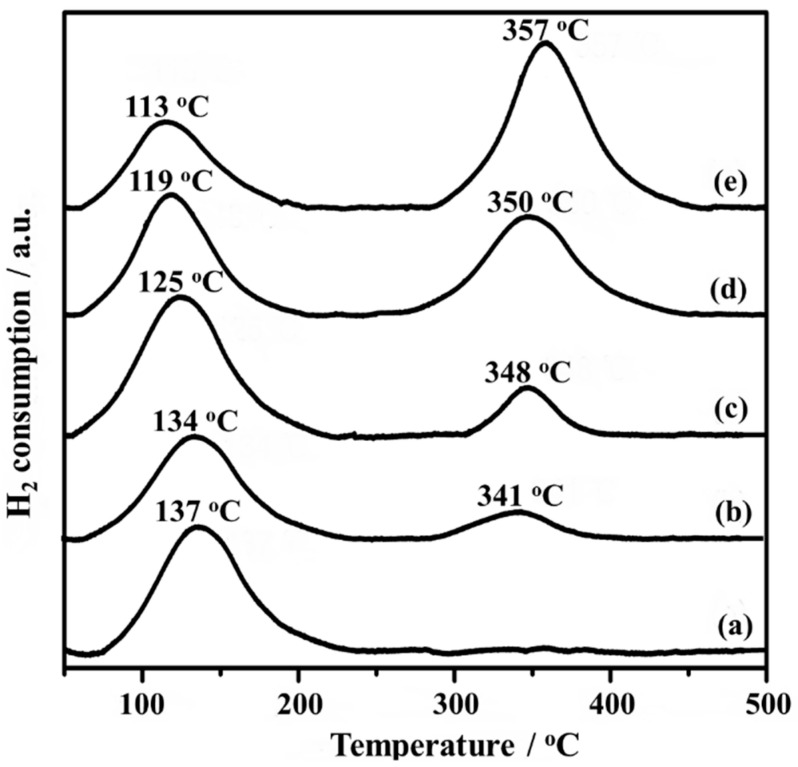
H_2_-TPR data of calcined (a) Ag/SiO_2_, (b) Ag-0.2%Ni/SiO_2_, (c) Ag-0.5%Ni/SiO_2_, (d) Ag-1%Ni/SiO_2_, and (e) Ag-3%Ni/SiO_2_.

H_2_-TPR was used to study the effect of the Ni content on the redox properties of calcined Ag-based catalysts (Figure 5). For Ag/SiO_2_, only one H_2_ consumption peak could be observed at 137 °C, corresponding to the reduction of a small amount of silver oxides to metallic Ag [5,13]. However, a new H_2_ consumption peak appeared in Ag-Ni/SiO_2_ catalysts at ca. 350 °C, which was ascribed to the reduction of nickel oxide species [5,32]. Compared with Ag/SiO_2_, the reduction peaks of silver oxide in Ag-Ni/SiO_2_ catalysts shifted to a lower temperature. Specifically, a small amount (0.2 wt.%) of Ni species introduced into Ag-Ni/SiO_2_ exerted a slight impact on the reduction behavior of silver oxide. With the increment of Ni loading from 0.5 wt.% to 3.0 wt.%, the center of the reduction peak shifted from 134 to 113 °C. These results revealed that after the incorporation of Ni, the interaction between the sliver oxide with silica was disturbed, and the reduction of silver oxide species was promoted, which also confirmed the interaction between Ag and Ni species [5]. In contrast, the reduction peaks of NiO shifted from 341 to 357 °C with the Ni loading varying from 0.2 wt.% to 3 wt.%, which may be attributed to the presence of more NiO.

**Figure 6:**
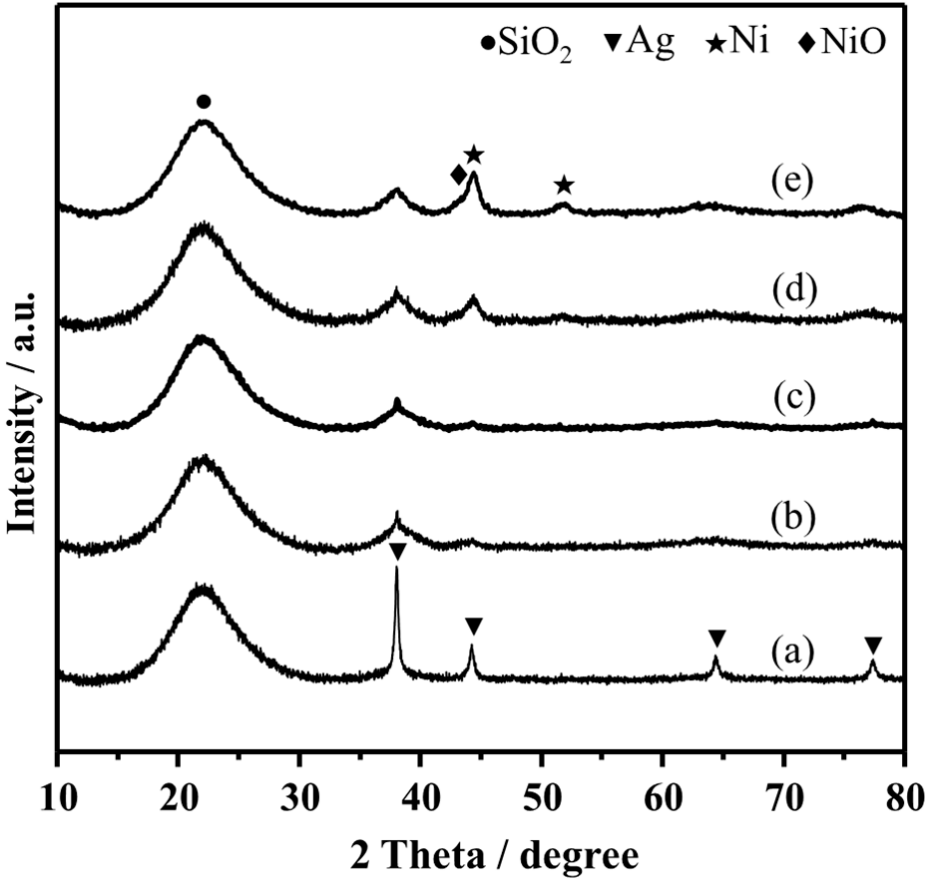
XRD patterns of reduced catalysts: (a) Ag/SiO_2_, (b) Ag-0.2%Ni/SiO_2_, (c) Ag-0.5%Ni/SiO_2_, (d) Ag-1%Ni/SiO_2_, and (e) Ag-3%Ni/SiO_2_.

The XRD patterns of the reduced catalysts were collected. As shown in Figure 6, for Ag/SiO_2_, four obvious peaks at 2*θ* = 38.1°, 44.3°, 64.4°, and 77.4° can be assigned to the (111), (200), (220), and (311) crystal planes of metallic Ag (PDF # 87-0597) [15], indicating that Ag species exist in the form of metallic Ag on the reduced catalyst. Notably, broader Ag peaks can be detected in Ag-Ni/SiO_2_ catalysts, suggesting that the introduction of Ni to Ag/SiO_2_ could enhance the dispersion of Ag nanoparticles. On the other hand, when the loading of Ni was lower than 1.0 wt.%, no characteristic peaks of Ni species could be detected. This result is possibly due to the presence of highly dispersed Ni species and/or the content of Ni was low and not sufficiently large enough to be detected by XRD. With increasing the Ni loading, the peaks of metallic Ni (44.3° and 51.7°) first appeared in Ag-1%Ni/SiO_2_ and became more obvious when the loading of Ni was increased to 3.0 wt.%. Additionally, Ag-1%Ni/SiO_2_ and Ag-3%Ni/SiO_2_ showed a weak shoulder peak at 43.3° corresponding to NiO, indicating that there was still a small amount of NiO in the reduced samples. A broad and faint peak at about 22° (due to amorphous SiO_2_ [33]) can be observed in all the catalysts.

The Ag crystallite sizes of the reduced catalysts were obtained according to the Scherrer equation. The average crystalline sizes of Ag in the Ag-Ni/SiO_2_ catalysts (3.9–4.7 nm) are smaller than that in Ag/SiO_2_ (16.9 nm). Specifically, with a Ni loading no higher than 0.5 wt.%, the particle sizes of Ag distinctly decreased from 16.9 to 3.9 nm, whereas further rising Ni loading in the range of 0.5–3 wt.% resulted in a gradual increase in the particle sizes of Ag from 3.9 to 4.7 nm. Evidently, Ag-0.5%Ni/SiO_2_ exhibited the smallest particle sizes herein. The results demonstrated that the introduction of Ni in Ag-Ni/SiO_2_ could improve the dispersion of Ag, and the improvement extent was the largest when the Ni loading was 0.5 wt.%. Additionally, the size of most particles in TEM was smaller than that measured by XRD, which may be due to the aggregation of some particles caused by high-temperature reduction.

**Figure 7:**
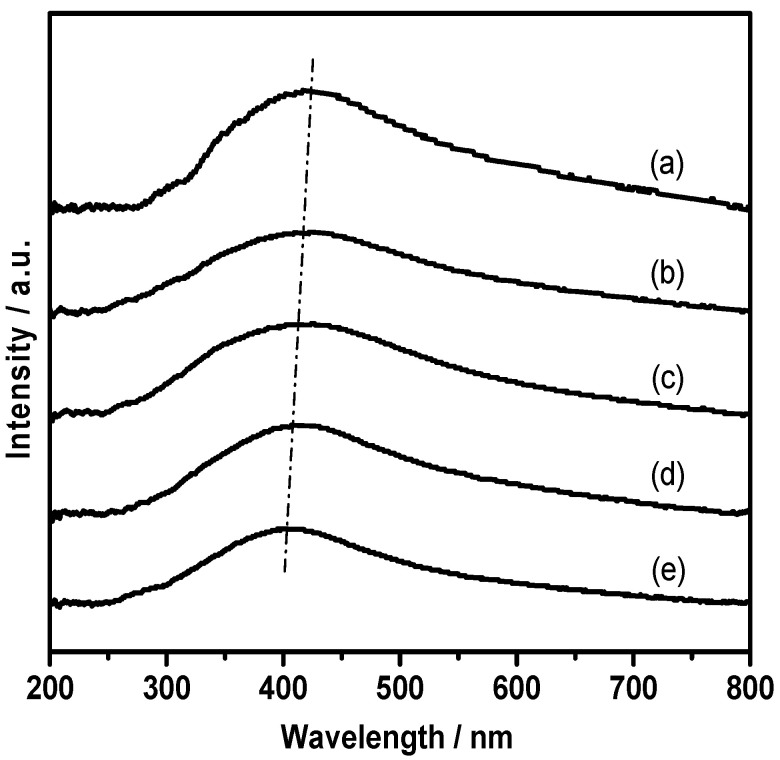
UV–vis DRS of as-reduced catalysts: (a) Ag/SiO_2_, (b) Ag-0.2%Ni/SiO_2_, (c) Ag-0.5%Ni/SiO_2_, (d) Ag-1%Ni/SiO_2_, (e) Ag-3%Ni/SiO_2_.

UV–vis DRS data of reduced catalysts were measured to elucidate the interaction between Ag and Ni. In Figure 7, Ag/SiO_2_ exhibited a broad peak centered at 421 nm, attributed to the surface plasmon resonance (SPR) band of Ag nanoparticles [34,35,36]. The maximum wavelength and width of the SPR are primarily dependent on the size and shape of the nanoparticles [36]. Meanwhile, the UV–vis DRS data of Ag-Ni/SiO_2_ catalysts also presented a single SPR band between 300 and 600 nm. No characteristic absorption band of Ni nanoparticles could be detected. However, there is some controversy in the literature about the optical absorption of Ni NPs. Xiang et al. [37] found the absorption bands at ca. 370 nm assigned to oxidized nickel nanoparticles and a broad absorption band at 550–700 nm assigned to nanostructures containing Ni^2+^ ions. Creighton et al. [38] demonstrated the calculated spectrum of Ni nanoparticles with an SPR in the 300–400 nm regions. Additionally, after incorporating Ni species, a blue shift for the absorption peak of Ag NPs was observed, implying that a chemical interaction occurred between Ag and Ni components, and the electronic properties of the bimetallic surfaces were changed [39,40].

**Figure 8:**
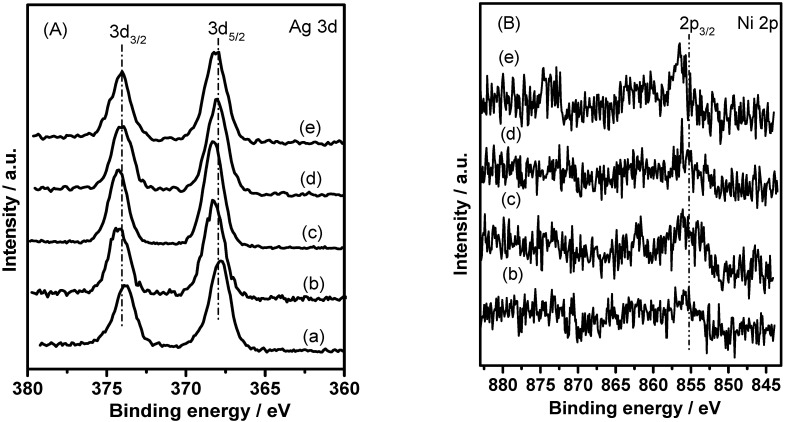
Ag 3d (**A**) and Ni 2p (**B**) photoelectron spectra of as-reduced catalysts: (a) Ag/SiO_2_, (b) Ag-0.2%Ni/SiO_2_, (c) Ag-0.5%Ni/SiO_2_, (d) Ag-1%Ni/SiO_2_, (e) Ag-3%Ni/SiO_2_.

XPS was employed to identify the chemical states and surface compositions of the reduced catalysts. For Ag/SiO_2_, the Ag 3d XPS peaks corresponding to the metallic Ag 3d_5/2_ and Ag 3d_3/2_ appeared at 367.8 and 373.9 eV, respectively (Figure 8A) [41]. Nevertheless, the BE of Ag 3d_5/2_ shifted to higher values with rising Ni concentrations, implying that the electronic effect was more obvious. The Ni 2p XPS spectra of the reduced Ag-Ni/SiO_2_ catalysts are shown in Figure 8B. The BE of Ni 2p_3/2_ was mainly at 856.1 eV which was assigned to Ni^2+^ species on the surface of the catalysts, and no characteristic peak corresponded to metallic Ni species (852.7 eV) [42]. This was probably because Ni was active, so metallic Ni on the surfaces of the reduced catalysts was easily oxidized to NiO during the sample installation operation in air [43].

**Figure 9:**
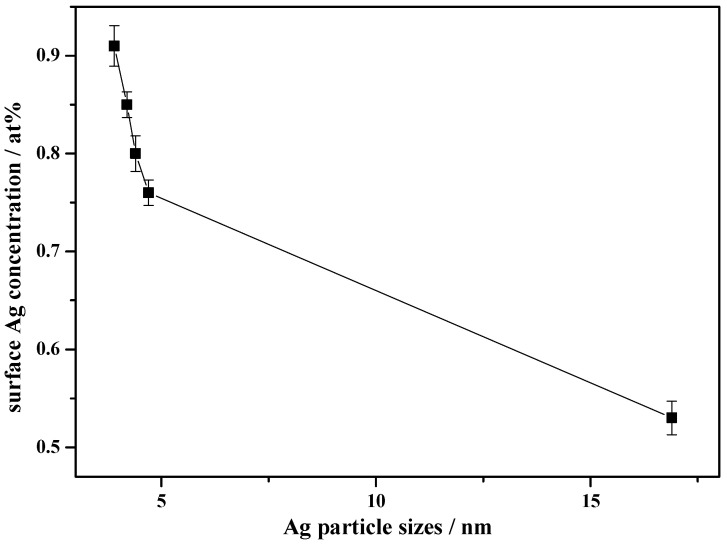
Surface Ag concentration as a function of Ag particle sizes for the different catalysts.

From the XPS results (Table 2), the Ag 3d_5/2_ peak of the reduced Ag-Ni/SiO_2_ catalysts shifted to relatively lower BE values compared with Ag/SiO_2_. Specifically, as the Ni content increases, the BE values of Ag species decreased from 368.5 to 368.0 eV, while the BE values of Ni species increased from 855.3 to 856.4 eV. These results showed that the charge transfer occurred between Ag and Ni species [5,32]. As shown in Figure 9, the surface Ag concentration (Table 2) increases gradually with the decrease in the Ag particle sizes (Table 3). The concentration of surface Ag reached the maximum when 0.5 wt.% Ni was loaded. When further increasing the Ni loading, the concentration of surface Ag decreased. Based on the deviation analysis, Figure 9 also illustrates that the difference in the Ag surface concentration was due to the different Ni concentration, not due to experimental incertitude. The above results clearly prove that for the supported catalysts with the same content of active component, the smaller size of the active component particles, the more active sites on the catalyst surface.

### 3.2. Catalytic Performance

**Scheme 1:**
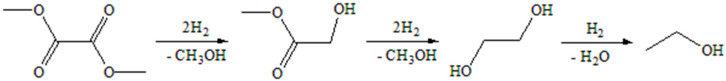
The reaction pathway for DMO hydrogenation.

As shown in Scheme 1, DMO hydrogenation includes DMO hydrogenation to MG, MG hydrogenation to EG, and EG hydrogenation to EtOH [5].

Under identical conditions for the evaluation of gas-phase DMO hydrogenation to MG, the catalytic performance of Ag/SiO_2_ and Ag-Ni/SiO_2_ was investigated. As shown in Table 4, both the DMO conversion and MG selectivity increased with the increase in Ni loading until they reached maxima at the same Ni loading of 0.5 wt.%. Therefore, Ag-0.5%Ni/SiO_2_ exhibited the highest MG yield (92.5%). This value is significantly higher than that of Ag/SiO_2_ (69.5%). We additionally prepared 0.5%Ni/SiO_2_, but it showed almost no catalytic activity in the reaction. For comparison, Zhou’s best Ag-Ni/SBA-15 catalyst showed 97.6% DMO conversion and 92.8% MG selectivity [5].

**Figure 10:**
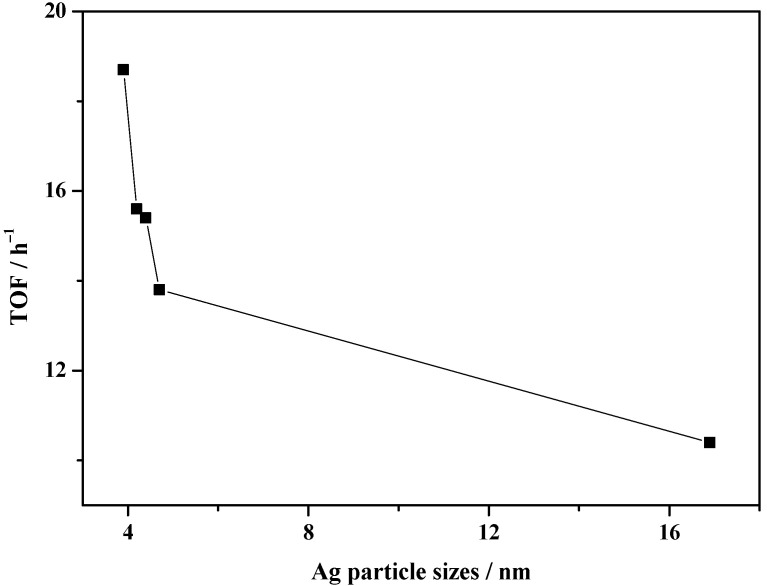
TOF values as a function of Ag particle sizes of the Ni-doped Ag/SiO_2_ catalysts.

To clearly compare the catalytic performance between Ag/SiO_2_ and Ag-Ni/SiO_2_ in DMO hydrogenation, the TOF values of all catalysts were measured at the conversion of DMO lower than 30% by regulating the DMO liquid hourly space velocity (LHSV) [13]. The DMO conversion data were used to calculate the TOF according to the active metal dispersion (Table 3). When increasing the content of Ni, both the TOF and *D*_Ag_ values increased first, passing through a maximum, and then decreased at higher Ni contents. Ag-0.5%Ni/SiO_2_ with the highest catalytic activity possessed a TOF of 18.7 h^−1^, superior to Ag/SiO_2_ (10.4 h^−1^). Moreover, the addition of Ni into Ag/SiO_2_ promoted the dispersion of Ag on SiO_2_, consistent with TEM results. The TOF values as a function of Ag particle sizes of the different catalysts are shown in Figure 10. The TOF value declines gradually with the increase in Ag particle size over these catalysts, suggesting that the DMO hydrogenation is a structure-sensitive reaction [4,16,26].

**Figure 11:**
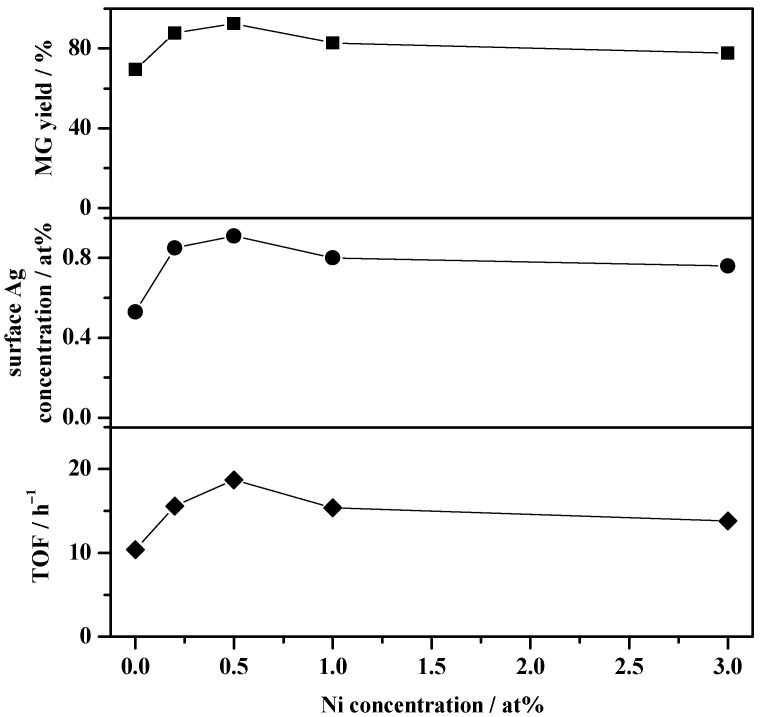
The influence of Ni content on MG yield, surface Ag concentration, and TOF values.

Generally, the changes in the structural properties and chemical states of active component might be the key factors affecting the catalytic behavior of Ag-based catalysts in DMO hydrogenation [19,27]. In our case, the MG yield of Ag-Ni/SiO_2_ catalysts is well consistent with the surface Ag concentration (Figure 11). In particular, Ag-0.5%Ni/SiO_2_ had the smallest Ag particle sizes and, hence, the largest number of exposed active sites, thus exhibiting the highest catalytic activity. Furthermore, the hydrogenation of DMO to MG is a structure-sensitive reaction on Ag-Ni/SiO_2_, and the intrinsic catalytic activity (TOF) increased with the decrease in Ag particle sizes (Figure 10). In addition, the MG yield of various Ag-Ni/SiO_2_ catalysts was in good agreement with the TOF values (Figure 11). In summary, it can be concluded that the addition of Ni improves the dispersion of Ag and reduces the Ag particle sizes, thus increasing the number of active sites and the specific activity of unit active site simultaneously, which ultimately improves the activity.

### 3.3. Catalyst Stability

**Figure 12:**
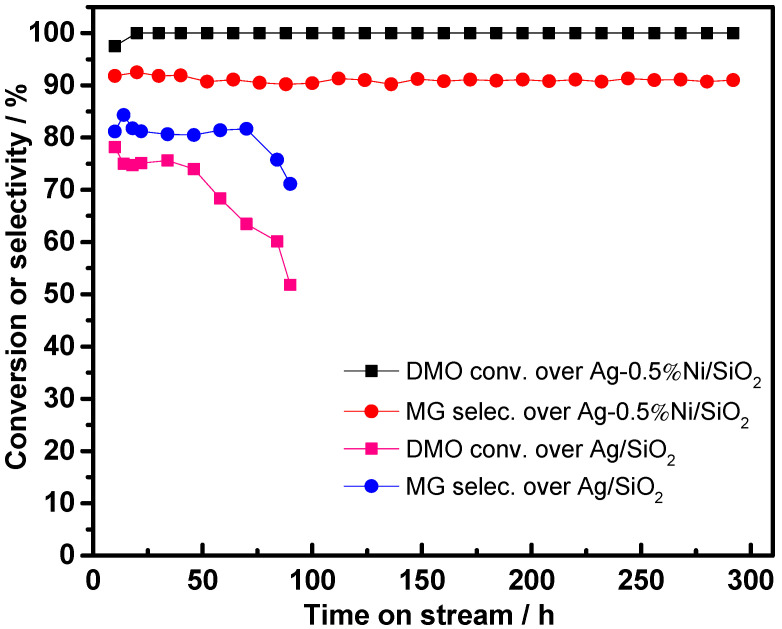
The long-term performance of the Ag/SiO_2_ and Ag-0.5%Ni/SiO_2_ catalysts. Reaction conditions: 493 K, 1.5 MPa, H_2_/DMO molar ratio = 150, LHSV = 0.28 h^−1^.

To investigate the effect of Ni on the stability, the long-term stability test of Ag-0.5%Ni/SiO_2_ and Ag/SiO_2_ was conducted under identical reaction conditions (Figure 12). Ag-0.5%Ni/SiO_2_ exhibited significantly improved performance in both DMO conversion and selectivity to MG, with no obvious changes, during catalytic reaction for 300 h. In contrast, Ag/SiO_2_ was distinctly deactivated within 90 h. Consequently, these results clearly proved that the stability of Ag catalysts could be improved distinctly by Ni doping.

**Figure 13:**
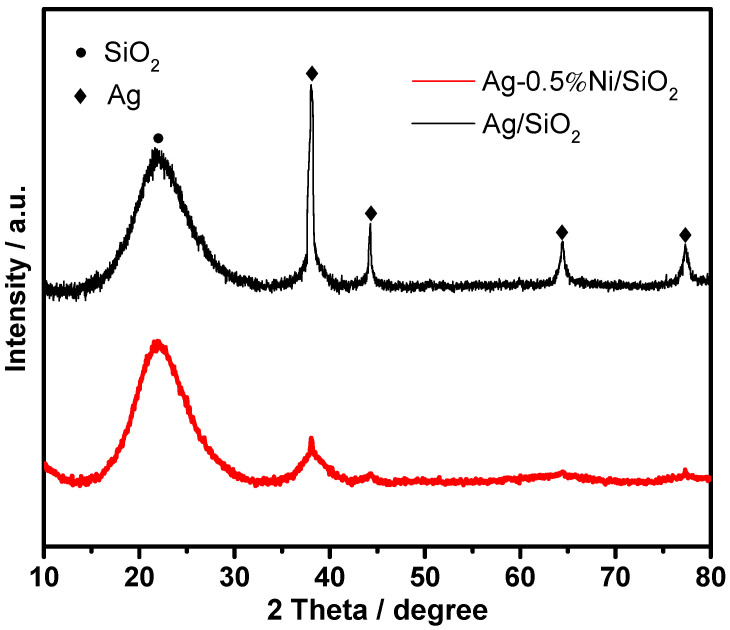
XRD patterns of the used Ag/SiO_2_ and Ag-0.5%Ni/SiO_2_ catalysts.

XRD patterns of the used catalysts were measured (Figure 13). Comparing the XRD patterns of Ag/SiO_2_ before and after the long-term performance testing, the average particle size of the deactivated catalyst increased to 18.9 nm, whereas that for the fresh catalyst was 16.9 nm (Table 3). For comparison, the size of Ag crystallites over Ag-0.5%Ni/SiO_2_ after 300 h reaction test was 4.1 nm, almost identical to that of the fresh catalyst (3.9 nm). Therefore, the deactivation of Ag/SiO_2_ was mainly caused by the growth of Ag particles, which resulted in the loss of active sites and the decreased TOF, as discussed above. Due to the weak interaction between Ag species and silica support, the Ag particles were prone to mobilize and accumulate to larger particles during calcination and reduction processes [44]. The extremely increased catalytic stability of Ag-0.5%Ni/SiO_2_ may be attributed to the stabilizing effect of the Ni species on the surface Ag species via strong interaction. According to the literature [5], the synergistic effect between Ni and Ag species prevented the surface transmigration of metallic Ag nanoparticles. Therefore, the aggregation of metallic Ag nanoparticles was limited after long-term testing. Similarly, Ni addition was also favorable for impeding the growth of Cu crystallites upon heat treatment [43].

## 4. Conclusions

The catalytic performance of a series of Ni-modified Ag/SiO_2_ catalysts for the hydrogenation of DMO to MG was investigated. Ag-0.5%Ni/SiO_2_ with a Ni content of 0.5 wt.% exhibited the best catalytic activity (complete conversion of DMO, 92.5% selectivity to MG). Moreover, Ag-0.5%Ni/SiO_2_ showed good stability on stream. The optimal Ni-modified Ag/SiO_2_ had higher dispersion of Ag species, more active sites, and higher sintering resistance due to the strong interaction between Ag and Ni species.

## Figures and Tables

**Table 1:** Physicochemical properties of calcined Ag/SiO_2_ and Ag-Ni/SiO_2_ catalysts.

Catalyst	*S*_BET_^a^ (m^2^∙g)	*D*_pore_^b^ (nm)	*V*_pore_^c^ (cm^3^∙g)	Ag Loading (wt.%)		Ni Loading (wt.%)	
				Theoretical	Actual ^d^	Theoretical	Actual ^d^
Ag/SiO_2_	179.4	10.6	0.65	10	9.8	0	-
Ag-0.2%Ni/SiO_2_	171.4	12.6	0.64	10	9.8	0.2	0.17
Ag-0.5%Ni/SiO_2_	161.1	12.0	0.60	10	9.9	0.5	0.46
Ag-1%Ni/SiO_2_	164.6	12.4	0.61	10	9.7	1.0	0.89
Ag-3%Ni/SiO_2_	153.9	12.6	0.60	10	9.8	3.0	2.85

^a^ BET specific surface area; ^b^ average pore diameter; ^c^ pore volume; ^d^ determined by XRF.

**Table 2:** XPS results for reduced Ag/SiO_2_ and Ag-Ni/SiO_2_ catalysts.

Catalyst	Binding Energy (eV)		Surface Elemental Concentration (at.%)	
	Ag 3d_5/2_	Ni 2p	Ag	Ni
Ag/SiO_2_	367.8	-	0.53	-
Ag-0.2%Ni/SiO_2_	368.5	855.3	0.85	0.26
Ag-0.5%Ni/SiO_2_	368.4	856.1	0.91	0.32
Ag-1%Ni/SiO_2_	368.1	856.2	0.80	0.89
Ag-3%Ni/SiO_2_	368.0	856.4	0.76	1.02

**Table 3:** TOF of Ag-Ni/SiO_2_ catalysts for DMO hydrogenation ^a^.

Catalyst	Conversion/%	Selectivity/%		d_Ag_^c^ (nm)	D_Ag_^d^/%	TOF_XRD_^e^/h^−1^
		MG	Others ^b^			
Ag/SiO_2_^f^	15.7	91.9	8.1	16.9	9.0	10.4
Ag-0.2%Ni/SiO_2_	24.7	95.6	4.4	4.2	28.0	15.6
Ag-0.5%Ni/SiO_2_	29.6	100	0	3.9	30.2	18.7
Ag-1%Ni/SiO_2_	23.2	97.6	2.5	4.4	26.8	15.4
Ag-3%Ni/SiO_2_	19.5	96.1	3.9	4.7	25.1	13.8

^a^ Reaction conditions: T = 493 K, P = 1.5 MPa, H_2_/DMO molar ratio = 150, LHSV = 1.73 h^−1^; ^b^ others include EG and MF; ^c^ average diameter of particle sizes of the reduced catalysts calculated by Scherrer equation; ^d^ dispersion of metallic Ag determined by XRD; ^e^ TOF_XRD_ was calculated by Ag dispersion; ^f^ LHSV = 0.58 h^−1^.

**Table 4:** Catalytic performance of Ag/SiO_2_ and Ag-Ni/SiO_2_ catalysts for DMO hydrogenation ^a^.

Catalyst	Conversion/%	Selectivity/%			MG Yield/%
		MG	MF	EG	
Ag/SiO_2_	77.8	89.3	2.2	8.5	69.5
Ag-0.2%Ni/SiO_2_	96.7	90.7	3.7	5.6	87.7
Ag-0.5%Ni/SiO_2_	100	92.5	0	7.5	92.5
Ag-1%Ni/SiO_2_	92.7	89.3	2.7	8.0	82.7
Ag-3%Ni/SiO_2_	87.2	89.1	5.0	6.0	77.7

^a^ Reaction conditions: T = 493 K, P = 1.5 MPa, H_2_/DMO molar ratio = 150, LHSV = 0.28 h^−1.^

## Data Availability

The data presented in this study are available in this article.

## Supplementary Materials

The following supporting information can be downloaded at: https://www.mdpi.com/article/10.3390/nano12030407/s1, Figure S1: Additional EDX-mapping images of Ag/SiO_2_ (a) and Ag-0.5%Ni/SiO_2_ (b).
