# Supplementary material for: Ni-Modified Ag/SiO2 Catalysts for Selective Hydrogenation of Dimethyl Oxalate to Methyl Glycolate
Source: Nanomaterials (Basel). 2022 Jan 26;12(3):407. doi: 10.3390/nano12030407 (PMC8838820; doi:10.3390/nano12030407)
Supplement: Supplementary file 1 [file nanomaterials-12-00407-s001.zip › nanomaterials-1496490-supplementary/nanomaterials-1496490-supplementary.pdf]

Supporting Information

Ni-Modified Ag/SiO_2 Catalysts for Selective Hydrogenation of Dimethyl Oxalate to Methyl Glycolate

Shuai Cheng [1], Tao Meng [1,*], Dongsen Mao [1,*], Xiaoming Guo [1], Jun Yu [1] and Zhen Ma [2]

[1] School of Chemical and Environmental Engineering, Shanghai Institute of Technology, Shanghai 201418, China; 156061301@mail.sit.edu.cn (S.C.); guoxiaoming@sit.edu.cn (X.G.); yujun@sit.edu.cn (J.Y.)

[2] Department of Environmental Science and Engineering, Fudan University, Shanghai 200438, China; zhenma@fudan.edu.cn

* Correspondence: mengtao@sit.edu.cn (T.M.); dsmao@sit.edu.cn (D.M.)

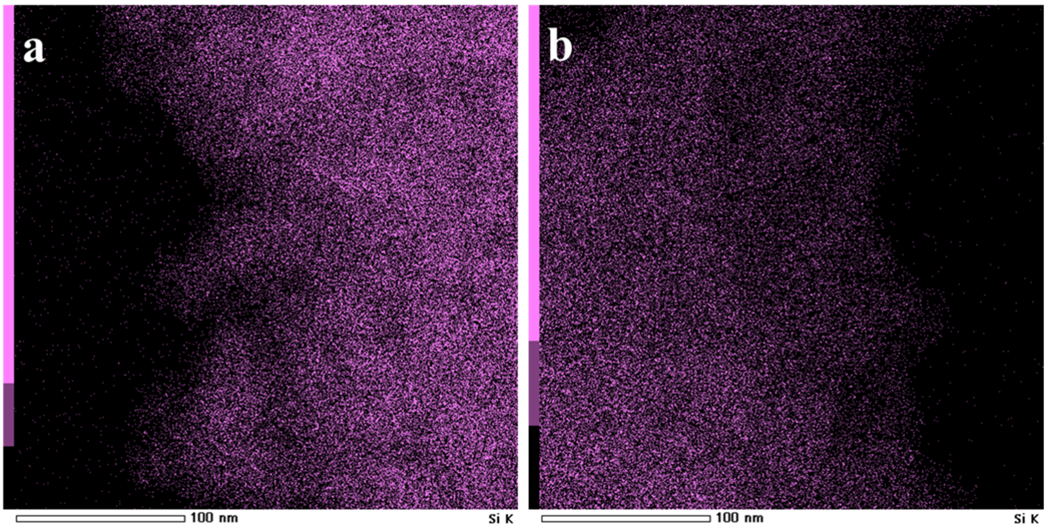

Figure S1. Additional EDX-mapping images of Ag/SiO_2 (**a**) and Ag-0.5%Ni/SiO_2 (**b**).
